# Physical function and pain after surgical or conservative management of multiple rib fractures – a follow-up study

**DOI:** 10.1186/s13049-016-0322-4

**Published:** 2016-10-28

**Authors:** Monika Fagevik Olsén, Margareta Slobo, Lena Klarin, Eva-Corina Caragounis, David Pazooki, Hans Granhed

**Affiliations:** 1 Department of Gastrosurgical Research and Education, Sahlgrenska Academy at Gothenburg University, Gothenburg, Sweden; 2 Department of Physical Therapy, Sahlgrenska University Hospital and Sahlgrenska Academy at Gothenburg University, Gothenburg, SE 413 45 Sweden; 3 Department of Surgery, Sahlgrenska University Hospital, Gothenburg, Sweden; 4 Department of Surgery, Sahlgrenska Academy at Gothenburg University, Gothenburg, Sweden

**Keywords:** Flail chest, Range of motion, Rib cage, Ribs, Spirometry

## Abstract

**Background:**

There is scarce knowledge of physical function and pain due to multiple rib fractures following trauma. The purpose of this follow-up was to assess respiratory and physical function, pain, range of movement and kinesiophobia in patients with multiple rib fractures who had undergone stabilizing surgery and compare with conservatively managed patients.

**Methods:**

A consecutive series of 31 patients with multiple rib fractures who had undergone stabilizing surgery were assessed >1 year after the trauma concerning respiratory and physical function, pain, range of movement in the shoulders and thorax, shoulder function and kinesiophobia. For comparison, 30 patients who were treated conservatively were evaluated with the same outcome measures.

**Results:**

The results concerning pain, lung function, shoulder function and level of physical activity were similar in the two groups. The patients who had undergone surgery had a significantly larger range of motion in the thorax (*p <* 0.01) and less deterioration in two items in Disability Rating Index (sitting and standing bent over a sink) (*p <* 0.05).

**Discussion:**

It is questionable whether the control group is representative since the majority of patients were invited but refused to participate in the follow-up. In addition, this study is too small to make a definitive conclusion if surgery is better than conservative treatment. But we see some indications, such as a tendency for decreased pain, better thoracic range of motion and physical function which would indicate that surgery is preferable. If operation technique could improve in the future with a less invasive approach, it would presumably decrease post-operative pain and the benefit of surgery would be greater than the morbidity of surgery.

**Conclusions:**

Patients undergoing surgery have a similar long-term recovery to those who are treated conservatively except for a better range of motion in the thorax and fewer limitations in physical function. Surgery seems to be beneficial for some patients, the question remains which patients.

**Trial registration:**

FoU i Sverige (R&D in Sweden), No 106121

## Background

Multiple rib fractures are a common and painful condition [1]. Greater work of breathing is required when several ribs are fractured, which can lead to respiratory failure, especially if the underlying lung parenchyma is injured [2, 3]. This is frequently the case in unstable thoracic cage injuries or “flail chest”, defined as three or more adjacent ribs each fractured in more than one location [4]. Flail chest can result in paradoxical chest movements and require ventilator support.

A number of new osteosynthetic implants specifically designed for stabilizing fractured ribs have been developed in recent years [5, 6]. Previous studies have shown that surgical stabilization of rib fractures can lead to decreased respiratory restrictiveness [7, 8]. The surgical management of unstable thoracic cage injuries may also decrease the need of intensive care and ventilator support with fewer complications, improved lung function and decreased overall cost [9–12].

However, there is a lack of prospective studies with a long-term follow-up concerning physical function, lung function, mobility and pain. We found in a previous study [13] that, of the 24 patients with multiple rib fractures who had undergone stabilizing surgery, 50 % still had pain after three months and 35 % after six months. Vital capacity was significantly decreased compared to normal values but there were no significant differences between the injured vs. non-injured side in breathing movements. Physical function was decreased with mild to moderate disability at three months and some to mild disability at six months.

There is a need for more trials with conservatively managed controls to further examine the effect of stabilizing surgery for rib fractures on long-term pain, physical function and lung function.

The aim of this follow-up was therefore to assess respiratory and physical function, pain, range of movement and kinesiophobia in patients with multiple rib fractures who had undergone stabilizing surgery and compare to conservatively managed patients.

## Methods

**Fig. 1:**
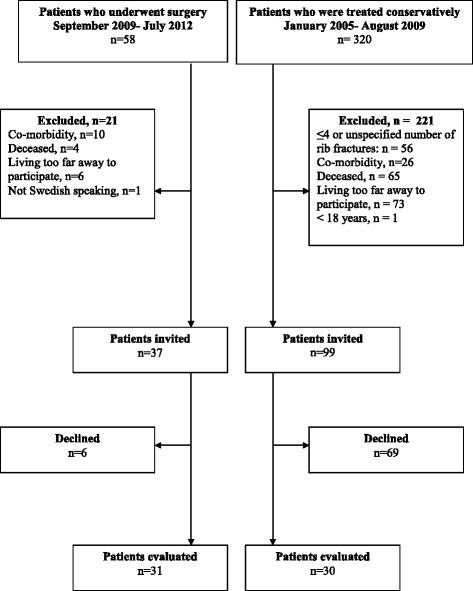
Flow chart

A consecutive series of 58 patients undergoing surgery at Sahlgrenska University Hospital due to multiple rib fractures during the period September 2010 to March 2012 were considered to participate in the follow up. The criteria for surgery were: (i) Flail chest defined as three or more adjacent ribs each fractured in more than one location, with respiratory insufficiency (ii) Multiple rib fractures (>4) with respiratory insufficiency and also in need of a thoracotomy due to bleeding or air leakage. Respiratory insufficiency was defined as failing arterial oxygenation despite oxygen administration. Patients with previous disease or trauma affecting lung function or range of motion in the rib cage (as chronic obstructive pulmonary disease, rheumatoid arthritis, stroke and major scoliosis), living too far from the hospital, <18 years or not Swedish speaking were excluded from the list. Of the remaining 41 patients, 4 were deceased, 6 declined so 31 were evaluated (Fig. 1).

To obtain a comparison group, a search was made in the trauma registry for patients with rib fractures due to trauma between January 2005 and August 2009. Three hundred and twenty patients were identified and their journals were scanned. Sixty-five of the patients were deceased, 56 were excluded as they had ≤4 or an unspecified number of rib fractures, and 100 fulfilled the above stated exclusion criteria leaving 99 patients who were invited by letter to participate (Fig. 1). Sixty-nine patients declined and 30 were included in the comparison. Demographic data on both groups are presented in Table 1. The mean age among the patients who had undergone surgery was 58.3 (range 23–88) years and in group who was conservatively treated 58.4 (range 23–87 years). There were no significant differences between the groups in demographic data except for a longer time since trauma in the comparison group.

**Table 1:** Demographic data (mean (±SD) or median (min-max) or number of patients)

	Stabilizing surgery (*n =* 31)	Conservatively treated (*n =* 30)	*p-*value
Sex, male/female	22/9	25/5	0.363
Age, years	58.3 (14.6)	58.4 (16.1)	0.908
Height, m	1.78 (1.09)	1.77 (0.09)	0.965
Weight, kg	81.4 (18.5)	85.6 (14.2)	0.175
BMI, kg/m^2^	25.5 (7.0)	27.4 (4.6)	0.231
Lung-disease, n (%)	2 (6 %)	3 (10 %)	0.671
Smoking history, yes/no/X	5/18/8	6/13/11	0.508
Time since trauma, years	1.8 (0.5)	4.5 (1.2)	<0.001
Number of ribs fractured, n	9 (4–20)	7 (5–13)	0.089
ISS, score	22 (9–48)	18.5 (9–45)	0.439

The patients who underwent surgery received an implant based on locked screws in low profile pre-shaped titanium plates (Matrix®) in order to stabilize the fractures. This system applies the concept of angular locked plates and intramedullary nailing and has been tested previously [14, 15]. A traditional thoracotomy was performed and the pleura cleaned, removing haematoma and debris when an injury to pleura, lung parenchyma or blood-vessels was suspected. When required, air and blood leakage were stopped and a resection of severely lacerated lung parenchyma was performed. Details about the surgery is presented previously [16]. Numbers of fractures stabilized was in average 4.8 (min 3 max 15) i.e. 56 % of the fractures were stabilized, but the flail segments were always stabilized, leaving a mechanically restored thoracic cage. The stabilizations were made with in median 6 plates (min 2- max 11) with additional fixation with cerclage and splints when limited access to the fractured area as under scapula. Sixteen patient (52 %) had lung injury of which one underwent a lobectomy, 8 resection of a segment (3 in combination with additional sutures) and 7 were only sutured. When m latissimus dorsi or m. serratus anterior were divided these were thoroughly sutured. The intercostal muscles were not sutured. Two chest tubes were inserted and kept for three to six days. Analgesia was individualized. Ropivacain was given in 25 of the 31 patients by a thin catheter placed in the pleura. Intravenous and oral morphine and paracetamol was given according to routines. None of the patients received epidural analgesia. Intravenous broad-spectrum antibiotic therapy was given to surgically managed patients until the chest tubes had been removed. Low-molecular weight heparin was given subcutaneously for a minimum of one week to prevent thrombosis. After surgery, the patients performed breathing exercises with positive expiratory pressure to increase lung volume. They were also mobilised as much as possible. They did not receive any specific breathing exercises or training to improve the range of motion at discharge from the hospital.

The patients who were treated conservatively were either admitted to the trauma ward or to the intensive care unit. In most cases, the indication for intensive care was the need for assisted ventilation in flail chest or concurrent injuries requiring support of vital functions. The majority of the patients received, according to routine, intravenous and oral morphine and paracetamol as analgesia. Six of the 30 patients received an addition of epidural anaesthesia with Bupivacain. A pain score <4 was aimed for when giving anaesthesia. Intravenous broad-spectrum antibiotic therapy was given if the patients had chest tubes and low-molecular weight heparin was given subcutaneously for a minimum of one week to prevent thrombosis. The patients received breathing exercises when indicated, based on low saturation or hyper secretion in the lungs. No other information or physical therapy treatment was given at the hospital or at discharge.

The following tests/assessments were undertaken at the follow-up:

### Pain

Intermittent or continuous persistent pain and pain during sleep and deep breathing was registered (Yes/No) and use of pain medication. The patients also had the possibility to note what aggravated the pain and what eased it (except pain medication).

### Spirometry

Forced Vital Capacity (FVC) and Peak Expiratory Flow (PEF) were tested in the sitting position in a standardized manner [17] according to the European Respiratory Society using an EasyOne spirometer (ndd Medical Technologies Inc.MA, US). The best value of at least three tests was recorded.

### Breathing movements

Breathing movements were tested by a Respiratory Movement Measuring Instrument, RMMI (ReMo Inc. Keldnaholt, Reykjavik, Iceland). The measuring device consists of six laser distance sensors with an accuracy of 0.0003 mm and a measuring frequency of 21Hz, an analogue to digital converter and a computer program for a PC computer. The equipment measures changes in distances between the diodes and the surface. In the tests, the diodes were placed bilaterally, at the level of costae 3, lower part of the thorax (xiphoid process) and abdominally (lateral to the umbilicus) with a distance of approximately 1/3 of the clavicle on each side of the thorax with the patient in the supine position [18]. Breathing movements were registered during breathing at rest and during maximal breathing movements. The movements were registered during one minute and the average movement was calculated.

### Range of motion in the thorax

Thorax excursion was assessed using a tape measure (marked in mm) around the circumference at two levels. Upper thoracic excursion was measured at the level of the 4^th^ costae and lower thoracic excursion at the level of the xiphoid process [19, 20]. The tests were performed standing with the hands placed on the head [19]. In order to be able to measure the maximal movements, instructions were given as follows: ‘Breathe in maximally and make yourself as big as possible’ and ‘Breathe out maximally and make yourself as small as possible’ [21].

Thoracic flexion was assessed by measuring the distance between skin marks at the 7^th^ cervical spinal process and 30 cm below when the subject was standing erect and after maximal forward bending of the back and the neck [22].

Lateral flexion was measured at the level of the tip of the index finger on the thigh when standing erect and then in a maximal lateral bending position [22].

### Range of motion and functional movements in the shoulder

Active flexion and abduction in the shoulders were measured in the sitting position by a goniometer [23]. The Boström index was used to assess functional movements in the shoulder [24]. It includes five bilateral movements graded from 6 (normal function) to 0. Normal function is defined as 60 points bilaterally and 30 points unilaterally.

### Physical function and level of physical activity

Physical function was estimated using the Disability Rating Index (DRI) that includes 12 items covering activities from dressing and going for walks to lifting heavy objects and exercising. The item responses were rated on visual analogue scales [25]. High values indicated impaired physical function.

Physical level was estimated using a 6-graded scale [26], where low values indicate a sedentary and high values an active lifestyle.

### Kinesiophobia

The Tampa score was used to evaluate fear of movement and a level > 37 was defined as having kinesiophobia [27, 28].

#### Statistics

No power analysis was performed as previous studies are missing. The sizes of the groups are therefore based on results from a consecutive series of patients who had undergone surgery and historical controls, see Fig. 1.

SPSS version 15.0 was used for the statistical analyses. Differences in gender, lung disease, smoking history, pain estimation, physical function, activity level and fear of movement between the two groups were analysed with the Mann–Whitney *U* test, Chi 2 test and Fisher’s exact test. Differences in demographic data as age, height and weight, and breathing movements, spirometry and range of motion were analysed with the *t*-test. Statistically significant differences were set at a *p* value <0.05.

## Results

### Pain

Results on frequency and persisting/intermittent pain, use of pain medication and pain during maximal breathing are presented in Table 2. There were no significant differences between the groups, but there was a tendency toward lower pain in the group who had undergone surgery. Activities which the patients reported aggravated the pain were: increased activity, lifting heavy objects, lying and coughing. In addition, they noted that activities that eased it were: resting, exercising, meditation and drinking alcohol.

**Table 2:** Experienced pain reported by the patients who underwent stabilizing surgery or were treated conservatively

	Stabilizing surgery (*n =* 31)	Conservatively treated (*n =* 30)	*p-*value
No pain	21 (68 %)	15 (50 %)	0.253
Intermittent/continuous pain, n	6/4	10/5	0.108
Pain disturbing sleep, n	7	7	0.944
Pain medication, n	4	7	0.319
Pain during maximal breathing, n	1	2	0.389

### Spirometry

FVC was in mean 103 % of the predicted value (SD 20 %) in the group of patients who had undergone stabilizing surgery and 111 % (29 %) in the group who were treated conservatively. Corresponding figures regarding PEF were 107 % (29 %) vs. 104 % (30 %). Four patients who had undergone surgery and two patients treated conservatively had an FVC <85 % of the predicted value. There were no significant differences between the two groups.

### Breathing movements

Results of measurements of breathing movement of the injured and the non-injured sides are presented in Table 3. There were only minor differences between the sides. The difference between the injured and non-injured side in the lower thoracic level was significantly larger in the group who had undergone surgery (*p =* 0.002), but the other positions did not differ significantly.

**Table 3:** Breathing movements (Δ Differences between injured vs. non-injured side) and range of motion

		Stabilizing surgery	Conservatively treated	*p-*value
Breathing movements	Δ Upper thorax during rest, mm	0.15 (1.27)	0.10 (0.42)	0.856
	Δ Lower thorax during rest, mm	0.13 (1.02)	−0.02 (0.36)	0.451
	Δ Abdominally during rest, mm	−0.61 (1.90)	−0.01 (0.77)	0.136
	Δ Upper thorax during maximal breathing movements, mm	−0.39 (4.69)	−0.11 (1.91)	0.606
	Δ Lower thorax during maximal breathing movements, mm	4.98 (4.67)	−1.19 (2.04)	0.002
	Δ Abdominally during maximal breathing movements, mm	−0.41 (4.67)	−0.46 (2.58)	0.398
Thorax excursion	Upper level, cm	5.2 (2.1)	3.7 (1.8)	0.005
	Lower level, cm	4.3 (1.92)	4.3 (2.3)	0.944
Range of motion in the thorax	Thoracic flexion, cm	4.0 (1.8)	2.4 (0.8)	<0.001
	Thoracic extension, cm	2.5 (1.1)	1.3 (0.5)	<0.001
	Lateral flexion towards the injured side, cm	15.9 (5.0)	14.8 (5.6)	0.494
	Lateral flexion away from the injured side, cm	15.4 (5.1)	14.8 (5.8)	0.743
Range of motion in the shoulder	Flexion injured side, °	154 (31)	158 (37)	0.747
	Flexion non-injured side, °	154 (38)	163 (27)	0.341
	Abduction, injured side, °	159 (27)	161 (34)	0.846
	Abduction, non-injured side, °	156 (37)	165 (30)	0.373

Mean ± SD

### Range of motion in the thorax

Range of motion in the thorax is presented in Table 3. There were no significant differences between lateral flexion toward or from the injured side in either of the groups. Patients who had undergone surgery had a significantly greater range of motion in the thorax excursion (upper level), thoracic flexion and extension as compared to conservatively managed patients (*p <* 0.05).

### Range of motion and functional movements in the shoulder

Range of motion in the shoulders is presented in Table 3. There were no significant differences in flexion or abduction between the injured and non-injured side in either of the groups. The proportion of patients with normal function in the Boström index in the injured side or index in total was not significantly different between the group that had undergone stabilizing surgery and the group treated conservatively (*p =* 0.171 and 0.062).

### Physical function and level of physical activity

**Fig. 2:**
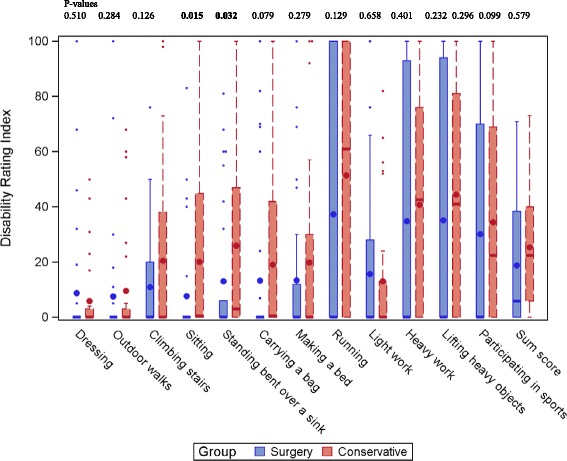
Boxplot of Disability Rating index by activity and group in patients who had undergone surgery or conservative treatment after multiple rib fractures. Large dot is mean, thick line is median and small dots are outliers

Results in DRI are presented in Fig. 2. The patients who had undergone stabilizing surgery had a DRI score of 19 mm in median and the patients treated conservatively 25 mm, implying mild to moderate disability. Ten of the items were scored higher in the group that was treated conservatively, but only two differences were significant, sitting and standing bent over a sink (*p <* 0.05). The patients in both groups scored their physical activity to median 4 (range 1–6) i.e. “moderate exercise 1–2 h a week, e.g. jogging or swimming or light physical activities more than 4 h per week”.

### Kinesiophobia

The proportion of kinesiophobia (Tampa score >37) was 32 % in the group that had undergone stabilizing surgery and 37 % in the group treated conservatively (N.S.)

## Discussion

The results of this study imply that patients who had undergone stabilizing surgery due to multiple rib fractures have less deterioration in their range of motion in the thorax and the thoracic spine than patients treated conservatively. In addition, patients who had undergone stabilizing surgery experienced significantly less limitation concerning sitting and standing bent over a sink. However, we were not able to detect any significant differences concerning pain, lung function, shoulder movement and physical function.

In this follow-up, 32 % of the patients who had undergone surgery and 50 % of those treated conservatively still had pain in the rib cage at the one-year follow-up. In our previous study of patients who had undergone stabilizing surgery due to multiple rib-fractures, [13] 52 % had remaining pain three months after the trauma and 35 % after six months. The proportion of patients with remaining pain is higher than previously reported, where 11 % had persistent pain after six months [8]. The high proportion of pain in conservatively treated patients in our study is notable but may also reflect that the patients who accepted to participate in the follow-up had remaining symptoms and were therefore more willing to participate. It is unknown what causes the chronic pain. Even though ribs are stabilized there can be additional, unremedied, minor fractures or fissures in the bone and cartilage of other ribs. It is therefore of importance to further study patients with remaining pain in order to find the cause of pain and give appropriate treatment.

Concerning VC and PEF, there were no significant differences between the groups and the proportion of patients within normal values was high even though nine of the patients undergoing stabilizing surgery had an additional resection of a lobe or segment. Due to the nature of the trauma, we have no lung function results from the patients before the surgery, but all values given are percent predicted [17]. In our previous study the patients had a forced vital capacity of 86 % of the predicted value in average after six months and a PEF of 77 %. The figures are also higher than those of Lardinois et al., who [8] found that 52 % had normal values (>85 % of predicted values) at six months after surgery. While the mean value in this current study is higher among those who underwent surgery, it is possible that lung function is restored over a longer period of time than six months and that different surgical techniques account for the difference in results [8, 13].

In the current study, the patients underwent registration of breathing movements with reliable and sensitive equipment [18, 29]. The results reveal that there are no major differences between the injured and non-injured side, neither after surgery nor after conservative treatment. Patients who had undergone surgery had even better breathing movements on the operated side of the thorax when measured in the level of the xiphoid process and significantly better than the conservatively managed group. Operated patients had also a significantly larger range of motion in the thorax. Campbell et al. [15] have undertaken a follow-up one to five years after stabilizing surgery, and 60 % of their patients reported chest wall stiffness. We did not specifically ask for stiffness in the thoracic cage but, according to the results of the measurement of thorax excursion, most patients were within reference values. However, the patients treated conservatively had significantly less range of motion in the thorax when measuring excursion, flexion and extension and the reason for this is unknown. It is possible that the fractures have healed with adhesion and fusion between the ribs.

Clinically we have noticed that some patients have a satisfactory range of motion but a decreased function in the shoulder after the operation, and we thus added such measurements to this protocol. However, we found no significant differences between the shoulders on the injured or non-injured side in either surgically or conservatively managed patients. The proportion of patients with a restricted range of motion in shoulder abduction after stabilizing surgery are in line with figures by Lardonois et al. [8]. A reason for a decreased range of motion after surgery may be the division of m. latissimus dorsi and m. serratus anterior when stabilizing the ribs under these muscles and trauma to motor nerves. Even though we did not find any significant differences between the injured and non-injured shoulder it is important to consider the trauma the surgery causes as how to perform the surgery by not only concerning the rib-fractures, but also how the incisions are made and weight the trauma of the surgery against the benefit of the procedure.

The patients in both groups are physically active to the same extent, but most of them less active than they desired. The question is whether the low activity level reflects a reduction after the trauma or whether the participants have a lower activity level in general. Patients in both groups had limitations in their physical function interpreted as a mild to moderate disability. It is always a challenge to evaluate patients after trauma as concurrent injuries may have an impact on activity and results concerning physical function. Collecting more information concerning activity and function previous to the trauma is thus of value for future trials.

In this survey we also included the Tampa score to be able to detect whether the patients experienced kinesophobia [26]. After trauma it is likely that the patients build up a fear of movement that can lead to kinesiophobia. About one third of the patients in both groups reached the level of 37 points, which is the cut-off level, and this is in line with previous trials of patients with orthopaedic injuries [28].

The study has several limitations. To compare two series of patients, one of which consists of historical controls is always a weakness. The time from trauma to follow-up differs between our two groups. The patients who were treated conservatively had been injured 2,5–6 years earlier compared to those who had undergone surgery 1–2.5 years earlier. The time-gap may have influenced the results as there have been staff changes and improvements in pain medication, and care during the period. Another difficulty of using historical controls is that the medical records are seldom complete. In our study we were not able to verify the number of patients with flail chest or with lung injury among the patients in the control group because of missing information and less detailed scans which is a limitation.

The two groups of patients in our follow up also received different regimens for analgesics. This may have had an impact of remaining pain, however, it is still unclear if pain is an indication to surgery in patients with multiple rib fractures but without flail chest. This follow up was further performed more than a year after surgery and the impact may be of minor importance. A limitation was also that the patients were only asked to assess level of remaining pain after the trauma and not the kind of pain to be able to understand the origin of it. As no radiography was performed at follow-up in order to diagnose non-union fractures it is difficult to distinguish between post-thoracotomy or post-trauma pain in the operated group. However, as there is a high incidence of pain in the conservatively managed group we interpret that the major part of this pain is due to the trauma.

It is questionable whether the control group is representative since the majority of patients were invited but refused to participate in the follow-up. On the other hand, the groups evaluated were equal in size and there were no major differences between the groups concerning demographic data and Injury Severity Score (ISS). Another limitation is that the group sizes were rather small to be able to detect significant differences in all the variables used in the study. The results therefore have to be interpreted with caution. The low number of patients also limited the possibility of further statistical analysis as multivariate analysis. In future studies, results from more than one centre may be necessary to be able to reach sufficient power to detect clinically important differences.

At the moment, most studies concerning surgery have focused on flail chest and respiratory insufficiency. Surgery seems to be beneficial for some patients, the question remains for which patients. Patients who are difficult to wean off a ventilator because of pain, those with lung injury and deformities of several ribs may be a specific group who may benefit from surgery. At our center, flail chest is an indication for surgery, however, it is still unclear if pain as an indication, with multiple rib fractures but without flail chest, should be operated. This study is too small to make a definitive conclusion if surgery is better than conservative treatment. But we see some indications, such as a tendency for decreased pain, better thoracic range of motion and physical function which would indicate that surgery is preferable. If operation technique could improve in the future with a less invasive approach, it would presumably decrease post-operative pain and the benefit of surgery would be greater than the morbidity of surgery.

## Conclusion

Patients undergoing surgery have a similar long-term recovery to those who are treated conservatively except for a better range of motion in the thorax and fewer limitations in physical function. Surgery seems to be beneficial for some patients, the question remains which patients benefit.

## Abbreviations

BMI: Body mass index
DRI: Disability rating index
FVC: Forced vital capacity
ISS: Injury severity score
PEF: Peak expiratory flow
RMMI: Respiratory movement measuring instrument
